# Characterisation of rare earth elements and toxic heavy metals in coal and coal fly ash†

**DOI:** 10.1039/d2ra02788g

**Published:** 2022-07-01

**Authors:** Ilemona C. Okeme, Richard A. Crane, William M. Nash, Theophilus I. Ojonimi, Thomas B. Scott

**Affiliations:** School of Physics, University of Bristol, HH Wills Physics Laboratory Bristol BS8 1TL UK ilemona.okeme@gmail.com +44 (0)7376625377; Camborne School of Mines, College of Engineering, Mathematics and Physical Sciences, University of Exeter UK; Environment and Sustainability Institute, University of Exeter UK; Department of Mining Engineering, University of Jos Jos 930222 Nigeria

## Abstract

Due to increasing demand for rare earth elements (REE), growing concerns over their sustainability, and domination of their supply by China, coal fly ash has recently emerged as a viable target for REE recovery. With billions of tonnes in repositories and still more being generated across the globe, it is necessary to develop environmentally friendly and economical extraction technologies for the recovery of the REEs from coal fly ash, and to consider the environmental implications of such a recovery process. This study reports characterisation of Nigerian simulant coal fly ash, and investigates the distribution and leaching of the REEs and U, Th, As, Cr, Cd and Pb from these materials using ethanoic acid. Significant amounts (14% to 31%) of the REEs were recovered in the acid-soluble fraction of a sequential extraction procedure using ethanoic acid. While the greatest amounts of U (53% to 62%) and Th (89% to 96%) were recovered in the stable residual fraction, significant amounts (3% to 13%) of U were recovered in the acid-soluble fraction. As was the most enriched element in the mobile acid-soluble fraction (46% to 60%), followed by Cd (15% to 34%). These results demonstrate that REEs contained within coal fly ash – especially those sourced from coal-fired power plants burning coal at temperatures between 700 °C and 1100 °C – can be recovered through an environmentally friendly procedure using the cost-effective heap leaching method, with ethanoic acid or the more cheaply-available vinegar as lixiviant. These results are also valuable for cost evaluation of rare earths recovery from coal fly ash generated by fluidised bed combustion coal fired power plants, and the development of methodologies for coal fly ash management.

## Abstract 2

Significant amounts of rare earth elements, REEs (14% to 31%) and critical REEs (16 wt% to 38 wt%) in the fly ash samples were recovered in the acid-soluble fraction of the sequential extraction procedure using ethanoic acid.

## Graphical abstract

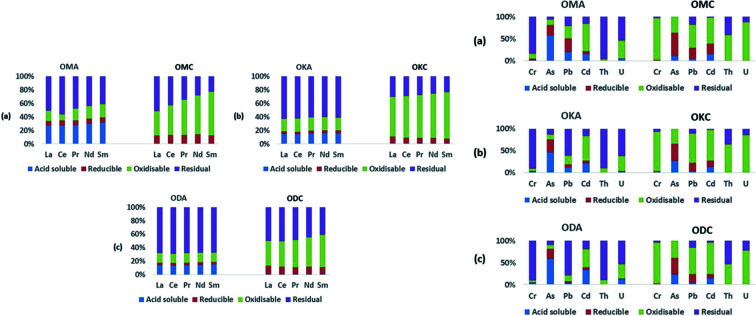

## 1. Introduction

The rare earth elements (REEs), often referred to as the ‘vitamins’ of modern industry, consist of the fourteen naturally occurring lanthanides, plus yttrium and scandium.^1,2^ Due to their unique or exceptional magnetic, electronic, catalytic and optical properties, the REEs are of vital industrial importance, with applications in the automotive, green technology, electronics and defence sectors.^3,4^ Since coming into the limelight in the year 2010, the demand for REEs has grown substantially over the past two decades due to their broad range of applications in advanced technologies.^5,6^

Currently, over 75% of REE production, and about half of the known reserves, are located in China.^7,8^ While there is a projected surge in demand over the coming decades for use in high-tech devices, there are growing concerns over REE sustainability, processing technologies, supply stability, geopolitics and trade policies.^4,9^ Consequently, there is a renewed stimulus for researchers and commercial institutions around the world to secure economically sustainable REE supplies, through research and development efforts focused on improving REE recovery methods, recycling, and alternate sources.^10^ Amongst these are a growing number of ‘unconventional sources’ of REEs, such as coal and coal fly ash.^11^

Several studies have been carried out on the viability of REE recovery from coal fly ash.^11–14^ These studies reported micron-sized REE-bearing minerals such as monazite, xenotime and zircon as the main REE minerals in the fly ash, which are encapsulated in the aluminosilicate glass that dominates fly ash composition. These REE-bearing minerals (originally present in the precursor coal) possess volatilisation temperatures considerably greater than those at which the coal is burnt, and therefore become concentrated in the fly ash generated by high temperature combustion.^15^ Coal fly ash has several advantages as a source of REEs: (i) it is cheap, abundant, and enriched in inorganic REE minerals such as phosphates; (ii) it is enriched in the heavy REEs (HREEs) which are the most limited in supply, rank highest in price, and are projected to increase in demand through the century; and (iii) extraction from fly ash need not involve the costly and energy intensive mining, crushing and grinding processes required by conventional REE ores.^16–20^ Despite these advantages of REE recovery from coal fly ash over the conventional ores, recovery of REEs from this material has been challenging. In addition to the comparatively low concentrations of REEs in coal fly ash (generally several orders of magnitude lower than those of conventional REE ores), several sequential extraction studies largely on coal fly ash sourced from pulverised coal combustion (PCC) power plants (operated at temperatures between 1300 °C and 1700 °C) have reported the REE-bearing minerals to be mostly concentrated in the residual fraction (>75%), entrapped in the difficult-to-leach glassy amorphous component of the coal fly ash.^16,21^ This glassy amorphous component of coal fly ash results from the high operating temperatures of coal fired power plants, which are significantly greater than the melting temperatures of clay minerals (such as kaolinite) in the precursor coal.^14^ Due to this entrapment, REE recovery from coal fly ash sourced from PCC power plants requires the use of costly and environmentally unfriendly extraction methods, such as acid extraction using sulphuric and hydrofluoric acids.^14^ These challenges have made commercialisation of promising laboratory-scale methods uneconomical due to their limited efficiency and/or high cost.^14,22–24^

Fluidised bed combustion (FBC) coal fired power plants operate at lower temperatures than PCC combustion plants (between 750 °C and 1000 °C), and sequential extraction studies on coal fly ash sourced from them have proven more effective at recovering metals.^25^ A study on fly ash sourced from a FBC coal-fired power plant and coal pre-treated at 600 °C reported a significant increase in leachability compared to coal fly ash sourced from PCB power plants.^25^ This was attributed to the formation of less significant amounts of glassy components and consequently non-entrapment of the REE-bearing microcrystals – a consequence of the lower combustion temperature, and the temperature being optimum for thermal decomposition of REE-bearing mineral microcrystals.

Although PCC power plants dominate globally, there are more than 6000 FBC power plants in operation (largely concentrated in China), with many more under construction or planned.^26^ With billions of tonnes of coal fly ash (from FBC coal fired power plants) already stored in repositories globally, and millions produced annually, sequential extraction studies to optimise and/or develop methodologies for REE extraction from this major untapped resource are urgently required.^27^

Another issue associated with coal fly ash and the extraction of REEs from it is the occurrence of toxic heavy metals – radiotoxic (U, Th) and chemotoxic (As, Cd, Cr, Pb), occurring as μm-sized particulates. These pose a human health and environmental hazard, making coal fly ash disposal a major concern.^28^ The hazard posed by these metals is dependent on their leachability, which depends on factors such as coal combustion temperature and varying natural environmental conditions such as surface- and groundwater redox potential and pH.^29^ An adverse combination of these factors can result in the toxic heavy metals leaching into water bodies, becoming mobile and bioavailable to plants and animals, and consequently to humans. Since the storage and disposal operations of materials that contain these elements are highly regulated, information on their leaching potential is vital for assessing the cost of extraction and separation of REEs from coal fly ash. Several studies on sequential extraction of toxic metals in fly ash sourced from both PCC and FBC power plants have been undertaken, and these indicate significant proportions (60% to 97% by mass) of the overall heavy metals to be associated with the stable residual fraction, and less significant amounts (<1% to 2%) to be associated with the mobile acid soluble fraction.^29–33^ To the extent that toxic heavy metals are associated with the mobile acid soluble fraction, this is attributable to the volatilisation and condensation of the metals onto the surfaces of more refractory coal fly ash particles during combustion, making them easily leachable.^33^

The present study investigates the sequential extraction of REEs and the toxic heavy metals U, Th, As, Cr, Cd and Pb from Nigerian coal (Omelewu coal (OMC), Okaba coal (OKC), Odagbo coal (ODC)) and simulant coal fly ash (Omelewu fly ash (OMA), Okaba fly ash (OKA), Odagbo fly ash (ODA)). It seeks to understand the partitioning and leaching behaviour of REEs and toxic trace metals, in order to assess the possible environmental impacts of REE recovery from these materials. This study builds on the findings of a previous analytical assessment of the REE concentration, distribution, speciation, crystallography and solid-state chemistry in the same simulant coal fly ash samples investigated here.^34^ In the previous study, the simulant coal fly ash samples OMA, OKA and ODA were found to contain total REE concentrations of 623 mg kg^−1^, 442 mg kg^−1^ and 441 mg kg^−1^ respectively. The mass fractions of the total REEs in each sample that are classified as critical (the elements Nd, Eu, Tb, Dy, Y, Er) in OMA, OKA and ODA were 43%, 34% and 33% respectively. Compared to bastnaesite ores – including the Bayan Obo deposit in China, which only yields about 10% critical REEs – these fly ash materials are enriched in critical REEs by three to four times.^16^

## 2. Experimental

### 2.1. Study area

**Fig. 1:**
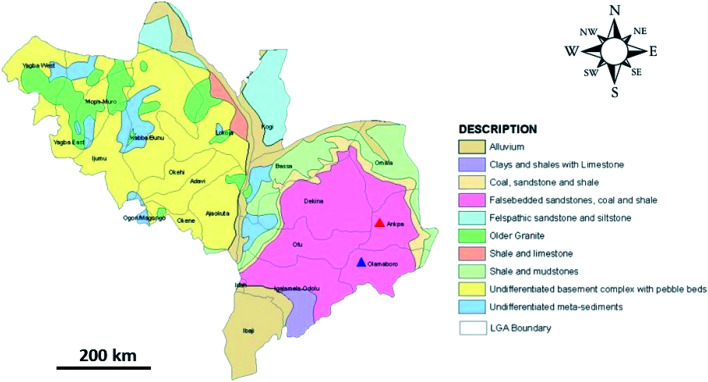
Geological map of Kogi state showing the location of Ankpa LGA (red triangle) and Olamaboro LGA (blue triangle).^42^

The coal samples characterised in this study were sourced from three open-pit coal mines located in Kogi state, Nigeria, shown in Fig. 1. The Okaba (OK) and Odagbo (OD) mines are located in Okaba and Odagbo respectively, within the Ankpa local government area (LGA). The Omelewu (OM) coal mine is located in Imane, within the Olamaboro LGA.

Kogi state lies at a latitude of 7.49 °N and longitude of 6.45 °E and is in the north-central geopolitical zone of Nigeria, with the confluence of the Niger and Benue rivers at its capital, Lokoja. Kogi state has an average maximum temperature of 33.2 °C and average minimum of 22.8 °C, with two distinct seasons: the ‘cold, windy and dusty’ dry season, which lasts from November to February; and a rainy season, that lasts from March to October. Annual rainfall ranges from 1016 mm to 1524 mm.^35^ The state experiences a tropical savannah (‘Aw’) climate according to the Köppen classification.^36^

Kogi state has a land area of 29 833 km^2^, with geological outcrops comprising two primary rock types; the significantly older basement complex rocks of Precambrian era in the western part of the state, and the cretaceous sedimentary rocks in the eastern part.^35^ The basement complex is made up of metasediments (migmatites, gneisses, schists, granites), and iron formations (rich in magnetite and haematite) with prominent outcrops south-east of Kabba city.^37^ The sedimentary formations in the eastern part of the state are divided into a number of sedimentary basins, namely; the Benue (central), Sokoto (north-west border), Chad (north-east), Bida (central), Dahomey (south-west) and Anambra (south-east) basins.^37^ The Anambra basin is mainly made of different formations, namely; the Nkporo, Mamu, Ajali and Nsukka formations.^38^ These formations are inter-bedded marine sandstones, siltstones, carboniferous-shale, coal and sandstones of a fluvial nature. These formations control the formation of coal, kaolin, clay, limestone, gemstones, slate, phosphate, gypsum and other associated minerals.^38^

The OK, OD and OM coal mines all host sub-bituminous coal (part of the Mamu formation) and belong to the Kogi mining district; a major coal resource within the Anambra basin, covering an area of 225 000 ha.^39–41^ The estimated reserves (in million tonnes) for OK and OD coal mines are 99 and 250, respectively.^40,41^ The estimated reserves for OM coal mine is unknown as little data are available.

### 2.2. Coal sample collection and preparation

In this study, coal samples were collected from each mine using a stratified random sampling methodology to ensure that samples collected were representative of the full compositional variability within the mine. This was achieved by dividing the coal field to be sampled into subareas followed by random sampling of each subarea at a distance of at least 200 m apart. A total of 15 coal samples per mine were collected. The raw samples (each approximately 1 kg in mass) were packed in polythene bags and transported to the UK for analysis.

Prior to technique-specific sample preparation, the individual coal samples were crushed and oven-dried at 100 °C for a period of 30 min to remove any moisture. The crushed samples were then pulverised and homogenised before being passed through a 150 μm wire mesh sieve. The crushing and pulverising was performed using an agate mortar and pestle.

For the various analyses, a composite coal sample was prepared per coal mine by mixing equal amounts of the 15 pulverised and homogenised coal samples. Similarly, a composite simulant fly ash sample was also prepared per coal mine.

#### 2.2.1. Simulant coal fly ash preparation

Since no operational coal-fired power plants yet exist in Nigeria (1200 MW of projected capacity remains at an advanced stage of planning), simulant coal fly ash samples were produced artificially and studied in this investigation.^43^ To simulate coal fly ash formation within the laboratory, sub-samples of each pulverised and sieved coal sample (between 200 g and 400 g in mass) were combusted in air using a Lenton™ ECF 12/6 muffle furnace at 1100 °C (below the fusion temperature of the ash, but sufficient to completely remove the organic matter). Each coal sample was heated from room temperature at a ramp rate of 10 °C min^−1^, the peak temperature (1100 °C) was maintained for 30 minutes, and the furnace was thereafter cooled to room temperature at a rate of 10 °C min^−1^. This peak combustion temperature approximates the temperature used in coal-fired power plants, especially for low-rank coal such as lignite and sub-bituminous coal, which is generally combusted in the 800 °C to 1200 °C range.^25,44^ An outcome of burning coal at a low temperature is that the rare earth and radioactive element bearing minerals are more likely to retain their identity as separate phases, rather than being encapsulated in the glassy component of coal fly ash, which don't melt at these reduced temperatures. Sieve analysis was performed using a set of sieves (45 μm, 75 μm, 150 μm and 300 μm) and a mechanical sieve shaker. Each composite sample was packed into the 300 μm sieve (with the other sieve sizes placed beneath), loaded onto the mechanical shaker, and vibrated for 3 hours to separate the particle size fractions. Results of the sieve analyses revealed that approximately 80% of the coal ash materials produced by the ashing process were in the particle size range of 1 μm to 300 μm (the typical range for fly ash). The synthesized materials thus comprised an 80% fly ash and 20% bottom ash mixture.

### 2.3. Analytical procedures

Scanning electron microscopy (SEM) and energy dispersive X-ray spectroscopy (EDS) were used to determine the identity of the minerals hosting the REEs and actinides in the coal and simulant fly ash samples, as well as the distribution of the REEs within these minerals. Sequential extractions were performed to determine the leaching potential of the REEs and toxic heavy metals in coal and simulant coal fly ash samples.

Preliminary proximate analyses, elemental composition and mineralogical analyses performed on the coal and simulant coal fly ash samples have previously been published.^34^ The simulant coal fly ash samples were shown to have a less complex mineralogy then their parent coal, being composed of quartz, mullite and cristobalite as the major mineral phases, with Pb, As, Cr, U and Th occurring only in concentrations from tens to hundreds of mg kg^−1^.^34^

#### 2.3.1. Scanning electron microscopy with energy dispersive spectroscopy (SEM-EDS)

Sample preparation and subsequent SEM-EDS analysis of the REE-bearing and actinide-bearing micro-mineral phases in the coal and fly ash samples have previously been published.^34^ SEM-EDS analysis showed the simulant coal fly ash samples to be composed of REE-bearing microcrystals of monazite, xenotime and zircon, and U-rich microcrystals with monazite the most predominant.^34^ In the current study, further SEM-EDS analysis of REE-bearing microcrystals in 3 subsamples each, from the composite simulant coal fly ash samples (OMA, OKA, ODA) is performed following the same procedure reported in the previous publication.^34^ EDS maps of the whole surface of each identified REE-bearing microcrystals were collected for 35 minutes to 50 minutes depending on the number of map slides.

#### 2.3.2. Sequential extraction

The sequential extraction procedure followed was the ‘Community Bureau of Reference’ (BCR) four-step sequential extraction method.^45^ Calibration was performed using an Inorganic Ventures ICP-71A multi-elemental ICP-MS calibration standard, which contained REEs, trace heavy metals and U and Th, among others. During sample analyses, blanks, duplicates and replicates of a United States Geological Survey (USGS) reference materials AGV-1 and DNC-1 were run every 10 samples to detect any instrumental drift and to verify the method's accuracy and reproducibility.

**Fig. 2:**
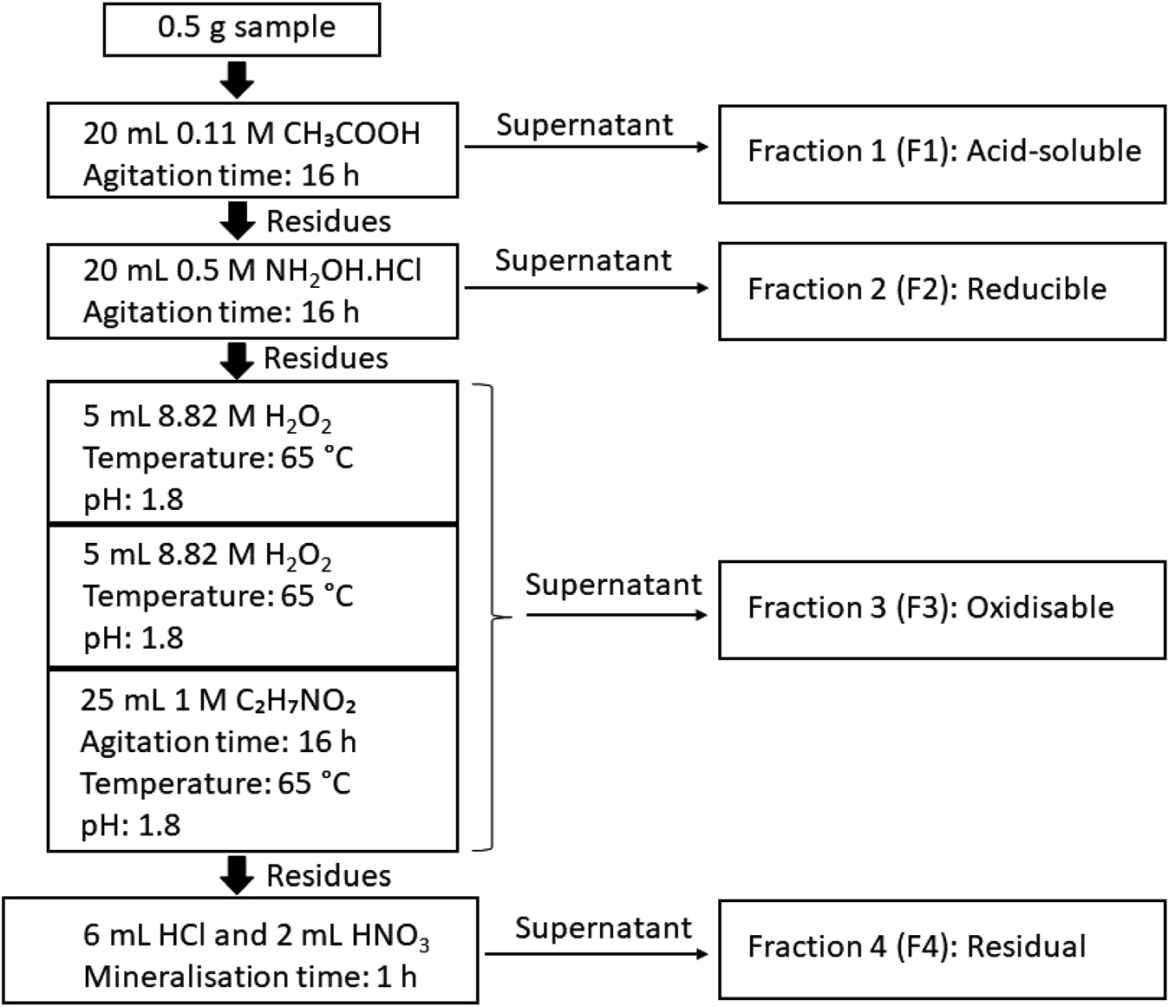
Schematic of BCR sequential extraction method.

The sequential extraction analysis in this study was performed on a total of six unsieved composite coal and fly ash samples (one composite coal sample and one composite fly ash sample per coal mine), namely the OMC, OKC and ODC composite coal samples, and the corresponding composite fly ash samples OMA, OKA and ODA. The sequential extraction procedure is described below and the schematic is shown in Fig. 2. Each step was completed in triplicate to assess reproducibility. An Agilent 7700x inductively-coupled plasma mass spectrometer (ICP-MS) was used to determine the concentrations of REEs, toxic heavy metals and the actinides U and Th in the sequential extraction leachates of the coal and simulant coal fly ash samples.

##### 2.3.2.1. Step 1: acid-soluble fractions

A 1 L solution of 0.11 M ethanoic acid (CH_3_COOH) was prepared by diluting 6.38 mL of concentrated ethanoic acid with MilliQ deionised water. Samples of 0.5 g mass were added to 50 mL centrifuge tubes, to which 20 mL of 0.11 M ethanoic acid was added using an auto-pipette. This was then loaded into a rotary mixer and mixed for 16 hours. The sample was then centrifuged at 3000 rpm for 2 minutes to accumulate a plug of solid material at the base of each centrifuge tube.

The supernatant from each was then filtered using 0.45 μm PTFE syringe filter, acidified using concentrated HNO_3_, and stored in plastic 50 mL flat-bottomed test tubes in a refrigerator. 10 mL of MilliQ water was added to each of the centrifuge tubes, and these were shaken vigorously to wash the solid residue. These residues were again centrifuged at 3000 rpm for 2 minutes, and the supernatant filtered using 0.45 μm PTFE syringe filters, acidified using concentrated HNO_3_, and stored in refrigerated plastic containers. Both supernatants were subsequently analysed independently. For each sequential analysis step and each fraction, the average of the analysis results for both supernatants was used for calculations.

##### 2.3.2.2. Step 2: reducible fractions

A 250 mL solution of 0.5 M NH_2_OH·HCl was prepared by combining 8.686 g of solid NH_2_OH·HCl salt with 250 mL of MilliQ deionised water in a volumetric flask. An auto-pipette was used to add 20 mL of 0.5 M NH_2_OH·HCl solution to each of the washed solids from step 1. As described in step 1 (acid-soluble fractions, Section 2.3.2.1), the same procedure for sample agitation, treatment of the residues and the preparation of both supernatants for analysis was applied in this step.

##### 2.3.2.3. Step 3: oxidisable fractions

A 250 mL solution of 1 M C_2_H_7_NO_2_ was made by dissolving 19.27 g of C_2_H_7_NO_2_ salt in MilliQ deionised water. This was stirred on a stirring plate, and its pH adjusted to 1.8 using concentrated HNO_3_. An auto-pipette was used to add 5 mL of 8.82 M H_2_O_2_ to each of the washed solids from step 2, which were put in a water bath at 65 °C until near dryness. A further 5 mL of 8.82 M H_2_O_2_ was added using an auto-pipette, and the samples were again left in a water bath at 65 °C, until near dryness. An auto-pipette was used to add 25 mL of the 1 M C_2_H_7_NO_2_ solution to each sample. Similarly, the same procedure for sample agitation, treatment of the residues and the preparation of both supernatants for analysis, described in step 1 (acid-soluble fractions, Section 2.3.2.1), was repeated in this step.

##### 2.3.2.4. Step 4: residual fractions

The solid remaining from the previous three steps was fully digested using aqua regia as follows. In polypropylene centrifuge tubes, 6 mL of concentrated HCl was added to each residual sludge sample. Once any reactions had subsided, 2 mL of concentrated HNO_3_ was added to each sample. Each centrifuge tube was covered with a watch glass and left for 15 minutes to allow the reaction to reach completion. The centrifuge tubes were then placed into a DigiPrep digestion block for 60 min at 95 °C. Once removed from the digestion block and allowed to cool, each sample was made up to a volume of 50 mL with MilliQ deionised water. The samples were filtered using 0.45 μm cellulose nitrate filters, after which they were diluted for ICP-MS analysis by adding 0.5 mL of the sample to 49.5 mL of 5% HNO_3_ (100× dilution).

## 3. Results and discussion

### 3.1. SEM-EDS analysis

**Fig. 3:**
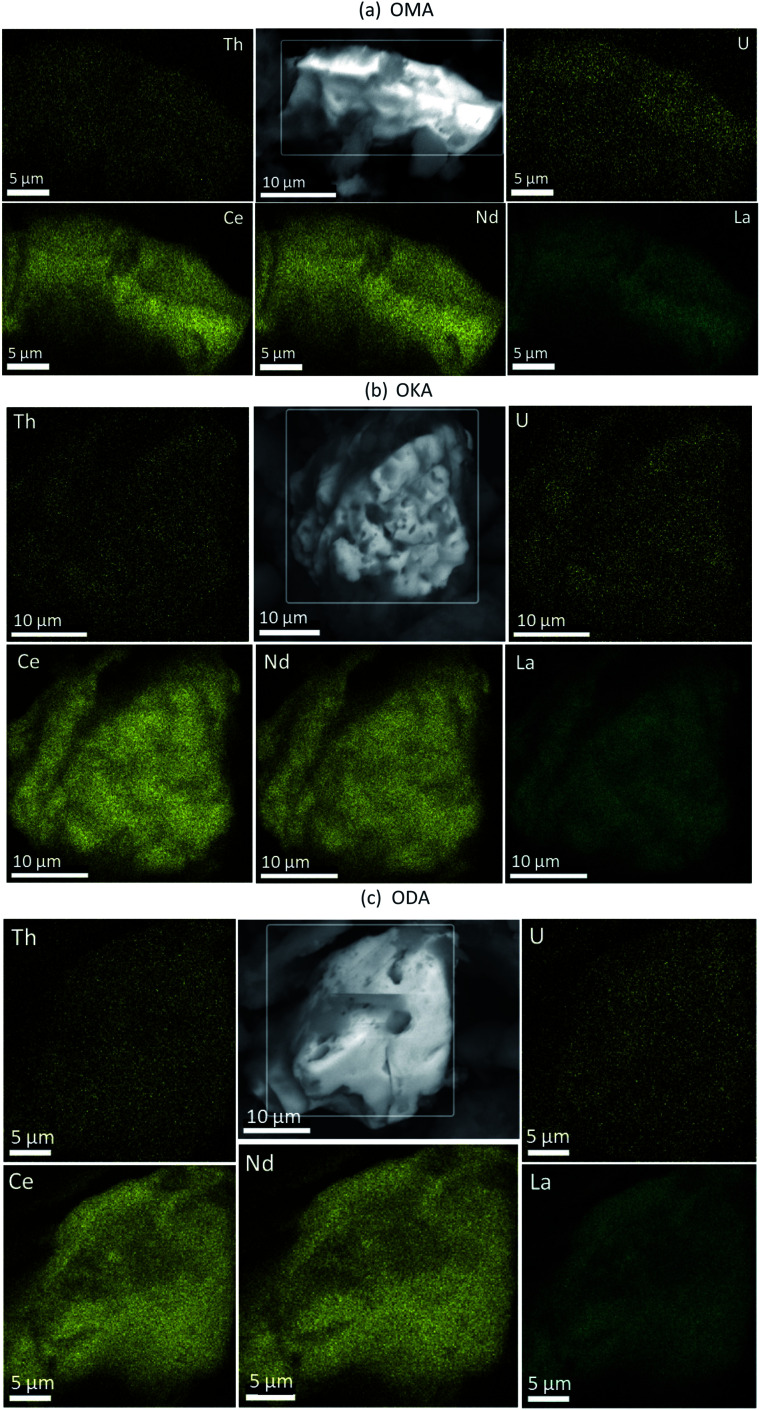
Backscattered electron image (grey) with EDS elemental maps of monazite particles in (a) OMA (b) OKA and (c) ODA, showing the distribution of Ce, Nd, La, Th and U.

**Fig. 4:**
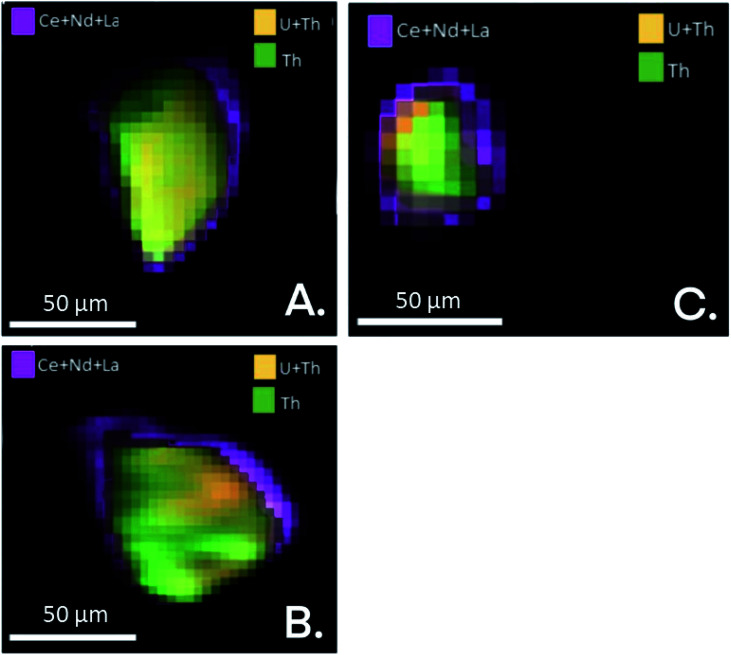
μ-XRF maps (Ce, Nd, La, U, Th) of monazite particles A, B and C, illustrating compositional variance of these elements. Modified from ref. 34.

Shown in Fig. 3 are the EDS maps of monazite microcrystals from the simulant fly ash samples OMA, OKA and ODA. These microcrystals are generally angular to sub-angular in shape, with weathering-induced pitted surfaces. The EDS maps reveal a homogenous distribution of REEs, U and Th; with the REEs being the more abundant. This is a possible indication that the REEs are distributed towards the surface of the monazite microcrystal, while U and Th occur deeper within it. This supports an earlier published synchrotron radiation μ-XRF analysis of monazite microcrystals (Fig. 4) isolated from the bulk simulant fly ash, which revealed a core–shell pattern, with the shell rich in Ce, La and Nd, and the core rich in Th and U.^34^ These results provide vital information which can subsequently be used to develop a more selective, cost-effective and environmentally friendly extraction methodology, targeted at the surface bound REE in fly ash monazite particles.

### 3.2. Sequential analysis

#### 3.2.1. REE fractionation

**Fig. 5:**
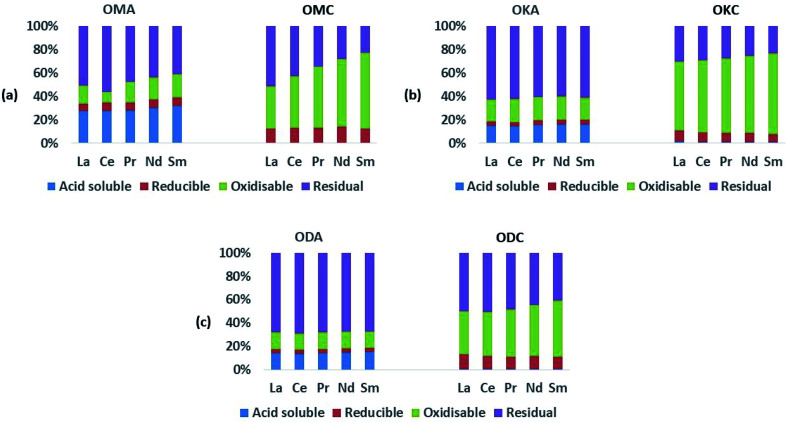
Results of sequential extraction for LREEs; (a) simulant coal fly ash (OMA) and the corresponding coal (OMC); (b) simulant coal fly ash (OKA) and the corresponding coal (OKC); (c) simulant coal fly ash (ODA) and the corresponding coal (ODC).

**Fig. 6:**
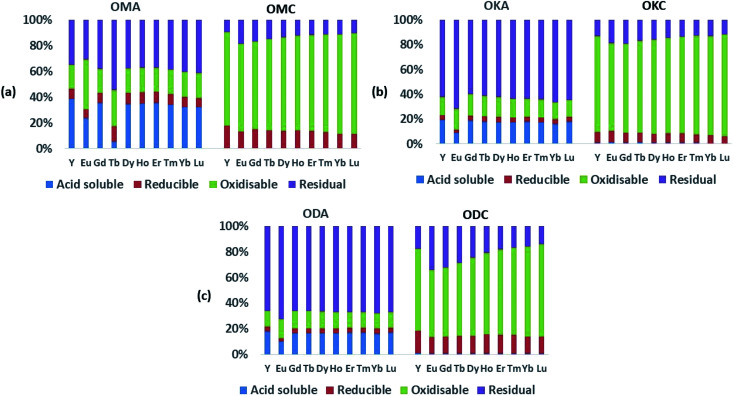
Results of sequential extraction for HREEs; (a) simulant coal fly ash (OMA) and the corresponding coal (OMC); (b) simulant coal fly ash (OKA) and the corresponding coal (OKC); (c) simulant coal fly ash (ODA) and the corresponding coal (ODC).

Fig. 5 and 6 show the results of sequential extraction for the light REEs (LREEs) and heavy REEs (HREEs) in the simulant coal fly ash (OMA, OKA, ODA) and coal (OMC, OKC, ODC) samples. The absolute values (mean ± standard deviation; in mg kg^−1^) of REEs in the various sequential extraction fractions for the simulant fly ash samples (OMA, OKA, ODA) are shown in Table 1, and the absolute values (mean ± standard deviation; in mg kg^−1^) of REEs in the coal samples (OMC, OKC, ODC) are shown in ESI Table S1.†

**Table 1:** Absolute values (mean ± standard deviation; in mg kg^−1^) of REEs in the various sequential extraction fractions for the simulant fly ash samples (OMA, OKA, ODA)^a^

Sample/fractions	La	Ce	Pr	Nd	Sm	Y	Eu	Gd	Tb	Dy	Ho	Er	Tm	Yb	Lu
**OMA**															
F1	5.57 ± 0.70	10.87 ± 0.70	1.56 ± 0.04	7.60 ± 0.20	1.67 ± 0.10	11.90 ± 0.80	0.33 ± 0.02	2.26 ± 0.10	0.03 ± 0.01	1.76 ± 0.10	0.35 ± 0.02	0.92 ± 0.01	0.11 ± 0.01	0.53 ± 0.08	0.08 ± 0.01
F2	1.15 ± 0.10	2.77 ± 0.10	0.38 ± 0.02	1.78 ± 0.10	0.39 ± 0.03	2.47 ± 0.20	0.10 ± 0.01	0.51 ± 0.03	0.08 ± 0.01	0.44 ± 0.03	0.09 ± 0.01	0.22 ± 0.02	0.03 ± 0.0	0.13 ± 0.01	0.02 ± 0.00
F3	3.18 ± 0.20	3.73 ± 0.20	0.98 ± 0.10	4.64 ± 0.30	1.04 ± 0.10	5.78 ± 0.50	0.54 ± 0.04	1.21 ± 0.10	0.17 ± 0.01	0.97 ± 0.10	0.19 ± 0.02	0.49 ± 0.04	0.06 ± 0.01	0.32 ± 0.03	0.05 ± 0.00
F4	10.24 ± 0.70	22.07 ± 1.70	2.65 ± 0.20	11.00 ± 0.80	2.16 ± 0.10	10.80 ± 0.80	0.43 ± 0.03	2.43 ± 0.20	0.34 ± 0.02	1.90 ± 0.20	0.37 ± 0.03	0.96 ± 0.06	0.12 ± 0.01	0.67 ± 0.04	0.10 ± 0.01
Total	20.14	39.44	5.57	25.02	5.26	30.95	1.40	6.41	0.62	5.07	1.00	2.59	0.32	1.65	0.25

**OKA**															
F1	5.02 ± 0.30	9.48 ± 0.50	1.18 ± 0.1	4.68 ± 0.2	0.91 ± 0.04	4.82 ± 0.3	0.14 ± 0.01	1.00 ± 0.01	0.14 ± 0.01	0.78 ± 0.04	0.15 ± 0.01	0.41 ± 0.02	0.05 ± 0.00	0.29 ± 0.02	0.05 ± 0.00
F2	1.17 ± 0.02	2.35 ± 0.02	0.27 ± 0.0	1.00 ± 0.01	0.20 ± 0.00	0.94 ± 0.01	0.03 ± 0.00	0.21 ± 0.00	0.03 ± 0.00	0.18 ± 0.00	0.03 ± 0.00	0.09 ± 0.00	0.01 ± 0.00	0.07 ± 0.00	0.01 ± 0.00
F3	6.48 ± 0.30	13.04 ± 1.0	1.50 ± 0.1	5.73 ± 0.3	1.06 ± 0.05	3.53 ± 0.2	0.27 ± 0.02	0.94 ± 0.04	0.13 ± 0.01	0.72 ± 0.03	0.13 ± 0.01	0.34 ± 0.02	0.04 ± 0.00	0.25 ± 0.02	0.04 ± 0.00
F4	21.17 ± 0.90	41.10 ± 2.4	4.57 ± 0.2	17.32 ± 0.7	3.42 ± 0.1	15.56 ± 0.8	1.11 ± 0.07	3.24 ± 0.1	0.48 ± 0.02	2.80 ± 0.1	0.56 ± 0.02	1.50 ± 0.09	0.20 ± 0.01	1.21 ± 0.07	0.16 ± 0.01
Total	33.84	65.97	7.52	28.73	5.59	24.85	1.55	5.39	0.78	4.48	0.87	2.34	0.30	1.82	0.26

**ODA**															
F1	4.87 ± 0.1	9.17 ± 0.06	1.12 ± 0.00	4.43 ± 0.03	0.90 ± 0.00	4.45 ± 0.04	0.17 ± 0.00	0.94 ± 0.00	0.14 ± 0.00	0.76 ± 0.00	0.15 ± 0.00	0.40 ± 0.00	0.05 ± 0.00	0.30 ± 0.00	0.04 ± 0.00
F2	1.05 ± 0.07	2.11 ± 0.14	0.24 ± 0.02	0.93 ± 0.06	0.19 ± 0.01	0.87 ± 0.05	0.04 ± 0.00	0.19 ± 0.01	0.03 ± 0.00	0.16 ± 0.00	0.03 ± 0.00	0.09 ± 0.00	0.01 ± 0.000.00	0.07 ± 0.00	0.01 ± 0.00
F3	5.04 ± 0.26	9.36 ± 0.23	1.15 ± 0.06	4.41 ± 0.23	0.85 ± 0.05	3.09 ± 0.14	0.24 ± 0.01	0.77 ± 0.03	0.11 ± 0.00	0.60 ± 0.03	0.11 ± 0.00	0.29 ± 0.01	0.04 ± 0.00	0.22 ± 0.01	0.03 ± 0.00
F4	23.29 ± 0.7	45.63 ± 1.34	5.28 ± 0.15	20.23 ± 0.79	4.01 ± 0.17	16.42 ± 0.56	1.18 ± 0.06	3.74 ± 0.19	0.54 ± 0.03	3.05 ± 0.16	0.59 ± 0.03	1.58 ± 0.06	0.21 ± 0.00	1.26 ± 0.04	0.17 ± 0.01
Total	34.25	66.27	7.79	30.00	5.95	24.83	1.63	5.64	0.82	4.57	0.88	2.36	0.31	1.85	0.25

a F1: acid soluble fraction; F2: reducible fraction; F3: oxidisable fraction; F4: residual fraction.

##### 3.2.1.1. Coal samples

As expected, the absolute values of the REEs in the coal samples (ESI Table S1†) were generally lower than the values in the fly ash samples, which implies the REEs occur diluted in the coal samples due to the fact that, compared to coal fly ash, coal is largely made up of organic matter. Relative to the total REEs recovered in the fly ash samples, the total concentration of REEs recovered in the coal samples were all lower – being 1.5 to 3 times lower in OMC, 3 to 4 times lower in OKC and 2.5 to 3 times lower in ODC. While the LREEs and the HREEs were recovered to a limited extent in the initial acid-soluble fraction (∼1 wt%), they were mainly contained in the oxidisable fraction (42 wt% to 64 wt% for LREEs; 63 wt% to 77 wt% for HREEs), the residual fraction (27 wt% to 47 wt% for LREEs; 13 wt% to 22 wt% for HREEs), and the reducible fraction (8 wt% to 13 wt% for LREEs; 7 wt% to 14 wt% for HREEs). These results are presented graphically in Fig. 4. While the concentrations of both LREEs and HREEs in the residual and reducible fractions confirm the occurrence of rare earth minerals and possible adsorption of REEs onto Fe (hydr)oxides, the very high concentrations of both LREEs and HREEs in the oxidisable fraction indicate that REEs in the coal samples are significantly associated with sulphides and organic matter. Studies have shown that coal organic matter may become REE-enriched during coalification, when REE-enriched leachates infiltrate the coal bed and become absorbed by the organic matter, and that since HREEs have a higher affinity for the organic matter, this explains their greater representation in the oxidisable fraction of the samples.^12,46,47^

It is notable that only a very small amount (∼1 wt%) of the critical REEs (Nd, Eu, Tb, Dy, Y, Er) were recovered in the exchangeable fraction for all coal samples, being mostly concentrated in the oxidisable fraction (52 wt% to 78 wt%), the residual fraction (10 wt% to 28 wt%) and the reducible fraction (7 wt% to 18 wt%). The implication of these results for REE recovery from pulverised raw coal is that REEs in coal cannot be easily recovered using ethanoic acid, as only ∼1 wt% of REEs will be recovered, with the remainder staying chemically locked inside the coal.

##### 3.2.1.2. Fly ash samples

In the fly ash samples (Table 1), the amounts of the REEs were significantly higher than in the coal samples. This was expected as the combustion process (*i.e.*, removal of the organic matter content) which generated the fly ash samples, concentrated the REEs in the fly ash. In the fly ash samples, both the LREEs and HREEs were mainly associated with the residual fraction (the overall range for both groups was 39 wt% to 68 wt%), confirming the SEM-EDS results presented in Section 3.1. However, significant amounts of both were associated with the acid-soluble fraction (14 wt% to 31 wt%). The proportions of both LREEs and HREEs in the oxidisable fraction (13 wt% to 22 wt%) were comparable to the acid-soluble fraction, and the least proportions were in the chemically reducible fraction (3 wt% to 8 wt%). These data are presented graphically in Fig. 5 and 6. While REEs in the residual fraction were attributed to a combination of REE-containing silicate minerals and rare earth metal oxides (formed during decomposition of REE-bearing organic matter in the coal during combustion), REEs in the oxidisable fraction result from the REE-bearing sulphide minerals.^48^ Compared with the mean values of REEs from the total dissolution analysis, the total values of REEs extracted in the sequential analysis (Table 1) translates to 24 wt%, 44 wt% and 45 wt% percent recovery for OMA, OKA and ODA, respectively, which indicate that significant amounts of REEs were retained in the residual fractions, most probably associated with resistate REE-bearing microcrystals such as monazite.^34^

The proportions of the critical REEs (16 wt% to 38 wt%) recovered in the acid-soluble fraction were significant and comparable to the amounts of the LREEs and HREEs in the acid-soluble fractions of all the fly ash samples, except for Tb (5 wt% to 18 wt%) and Eu (9 wt% to 23 wt%). The significantly high amount of REEs recovered in the acid-soluble fractions is attributable to the occurrence of easily soluble calcium oxide, periclase, and other basic oxides (formed from the decomposition of REE carbonate minerals such as bastnaesite and synchysite) – although not detected in this study, previous studies have detected REE-bearing oxide of Ca in coal fly ash.^48^ Also, the significantly high amounts of REEs recovered in the acid-soluble fractions attributable to increased solubility of difficult-to-leach rare earth minerals due to thermal decomposition of their matrix during combustion, while also reducing the formation of glassy phases and encapsulation of REE minerals in such phases. A previous study of fly ash sourced from fluidised bed combustion (FBC) coal-fired power plant (operated at between 750 °C and 900 °C) reported improved REE extractability due to thermal decomposition.^25^ This supports the finding in the current study, and demonstrates the metallurgical advantage of burning coal at low temperatures (below 1200 °C). Although most sequential extraction studies on coal fly ash from PCC power plants reported over 70 wt% of REEs to be in the insoluble residual fraction and less than 4% in the acid soluble fraction, the results from the present study on simulant coal fly ash indicate a more balanced distribution.^24,49,50^ The results presented herein agree more closely with the findings of Taggart *et al.*, whose sequential extraction study of PCC derived fly ash reported 14 wt% REEs recovery from the acid soluble fraction.^51^ They also reported that a high proportion of the REEs was recovered from the oxidisable fraction (16.9 wt%), which was attributed to a higher amount of unburnt carbon in the coal fly ash samples. The same study also trialled oxalic acid instead of acetic acid; a significantly higher REE recovery in the acid-soluble fractions was reported. However, this higher recovery of REE in the acid soluble fractions when oxalic acid was used did not appreciably increase the total of REEs (that is, the sum of all fractions) recovered, and was accompanied with lower REE recovery and a much higher recovery of the unwanted toxic heavy metals, both in the reducible fractions. The enhanced recovery of the toxic heavy metals is thought to be due to the metal chelating potential of oxalate (a dicarboxylate) compared to acetate (a monocarboxylate).

#### 3.2.2. Toxic heavy metal fractionation

**Fig. 7:**
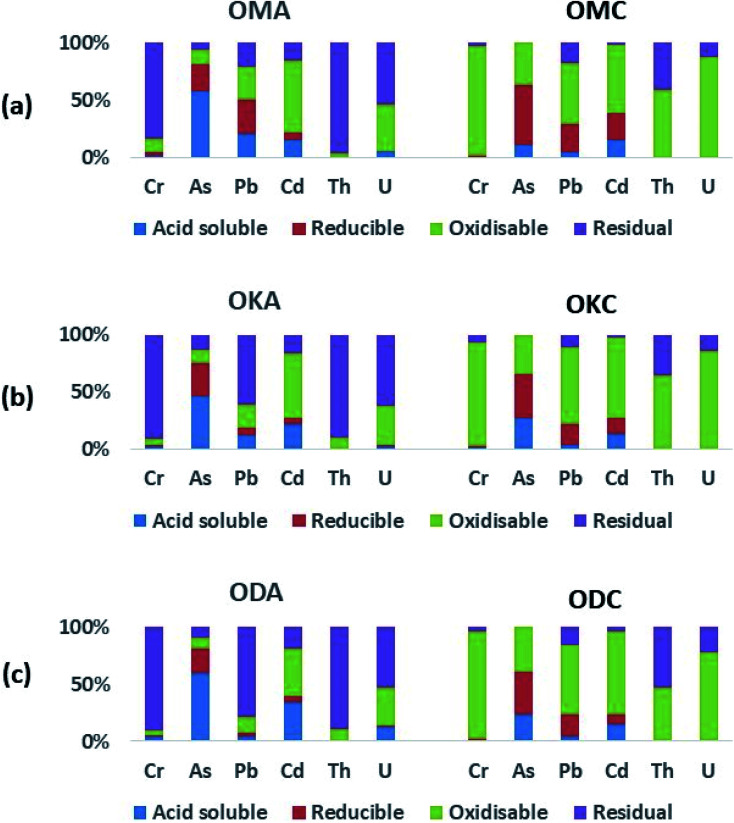
Results of sequential extraction of toxic heavy metals from coal and simulant coal fly ash. (a) Simulant coal fly ash (OMA) and the corresponding coal (OMC). (b) Simulant coal fly ash (OKA) and the corresponding coal (OKC). (c) Simulant coal fly ash (ODA) and the corresponding coal (ODC).

Fig. 7 shows the results of sequential extraction of the Cr, As, Pb, Cd, U and Th from coal and fly ash samples. The absolute values (mean ± standard deviation; in mg kg^−1^) of these toxic heavy metals in the various sequential extraction fractions for both coal and simulant fly ash samples (OMC, OKC, ODC, and OMA, OKA, ODA) are shown in ESI Table S2.†

##### 3.2.2.1. Coal samples

In OMC, in the acid-soluble fraction, only As (11 wt%), Pb (4 wt%) and Cd (15 wt%) were significantly recovered, with only very small amounts (∼1 wt%) of Cr, Th and U recovered in the same fraction. In the oxidisable fraction, Cr (96 wt%), Pb (53 wt%), Cd (60 wt%), Th (59 wt%) and U (87 wt%) were most recovered, with As less represented (37 wt%). This indicates high affinity of these toxic heavy metals for organic matter during coalification, occurring as coordination complexes (chelates) within the organic structure of the coal, as well as the occurrence of sulphide minerals of Pb (*e.g.* galena).^52^ While the amount of U recovered in the reducible fraction of OMC (0.3 wt%) indicates very little U adsorption onto the surface of (hydr)oxides of Fe and Mn, the high amount of U in the oxidisable fraction (87 wt%) indicates lower solubility (and therefore lower environmental mobility). As was most recovered in the reducible fraction (52 wt%), alongside significant amounts of Pb (25 wt%) and Cd (24 wt%). These results indicate: (i) occurrence of asenopyrite and pyrite in the coal samples; and (ii) to a lesser extent adsorption of As, Pb, Cd onto the surface of (hydr)oxides of Fe and Mn during coalification. Studies have shown that (hydr)oxides of Fe and Mn are good scavengers of toxic heavy metals.^52,53^ Th (41 wt%), Pb (18 wt%) and U (13 wt%) were also associated with the residual fraction, an indication of the occurrence of Th and U in silicate minerals (*e.g.* zircon, coffinite, thorite) alongside radiogenic Pb from the decay of Th and U.

In OKC and ODC, a similar distribution of the toxic heavy metals was recorded, with As distributions in the acid-soluble fractions of OKC (27 wt%) and ODC (23 wt%) being about two times higher than in OMC (11 wt%). These results indicate strong similarities between the mineralogy and geochemistry of OMC, OKC and ODC coal samples.

##### 3.2.2.2. Fly ash samples

The abundances of the toxic heavy metals in OMA, OKA and ODA were generally higher than those in OMC, OKC and ODC, due to the destruction of organic matter of the coal samples during combustion, and subsequent concentration of the heavy metals into the residual fly ash. The proportions of Cr recovered in the acid-soluble fraction for OMA (∼2 wt%), OKA (∼3 wt%) and ODA (∼4 wt%) were two to four times higher compared to OMC, OKC and ODC (in which ∼1 wt% Cr was recovered). In the acid-soluble fraction of the fly ash samples, As (46 wt% to 60 wt%) was the most enriched, followed by Cd (15 wt% to 34 wt%). In the acid-soluble fraction, the proportions of Pb recovered from ODA (4 wt%) was three to five times lower compared to its proportions in OMA (21 wt%) and OKA (13 wt%). The greatest proportions of U (53 wt% to 62 wt%) and Th (89 wt% to 96 wt%) were recovered in the highly immobile residual fraction, followed by the oxidisable fraction (34 wt% to 41 wt% for U; 4 wt% to 10 wt% for Th). Compared with OMC, OKC and ODC, (with 0.2 wt%, 0.1 wt% and 0.5 wt%, respectively, of U recovered in the acid-soluble fraction), U recovered in the same fraction from OMA (5 wt%), OKA (3 wt%) and ODA (13 wt%) was 25 to 30 times higher but still proportionately low. Compared to the U recovered in the other fractions, the amount of U in the acid-soluble fraction (being very mobile and soluble), although low, is of most concern for human health and the environment. In this regard these results agree with previous studies on fly ash sourced from a coal-fired thermal plant.^51,54^

### 3.3. Implications of REE and toxic heavy metals fractionation for REE recovery

These results of the percentage of REE recovered in the acid soluble fraction in all the fly ash samples imply that REEs (especially the critical REEs) can be recovered from fly ash using ethanoic acid. Compared to sulphuric and hydrochloric acids, ethanoic acid is biodegradable and environmentally friendly, and it is the main component of cheap and readily available vinegar. To maximise REE recovery from these fly ash materials while minimising costs, heap-leaching using ethanoic acid, is proposed.

The heap-leaching method is a low-cost industrial metal extraction process during which a large heap of (pulverised) ore or sample of interest is placed on an impermeable liner and treated with chemical solutions (‘lixiviants’), producing a metal-laden (‘pregnant’) leach solution.^55^ The dissolved REEs in the leach solution are then recovered *via* a selective extraction process such as the conventional solvent, liquid membrane or ion exchange extraction. Due to its low operating costs, minimal handling procedures, low energy requirement and low solvent consumption, the liquid membrane extraction (LME) process has been proposed as a more efficient and cost effective alternative to solvent extraction processes for the recovering REEs from coal fly ash.^56–58^ As the fly ash materials need no pulverisation, and only a readily available acid (ethanoic acid) is required to extract the REEs, this process is potentially highly cost-effective. Compared to REE recovery *via* total acid digestion of fly ash materials, the proposed process greatly reduces the toxicity of waste to be handled, which translates to lower cost. This proposed process of using ethanoic acid in a heap leach process is potentially suitable for the cost-effective recovery of significant amounts of REEs from a substantial proportion of the millions of tonnes of fly ash materials (generated from FBC coal-fired power plants) that exist globally.

From the perspective of health and environmental protection, As, Pb, Cd, Cr and U in the acid soluble fraction are of serious concern, due to their high mobility, solubility and bioavailability, as because these metals are carcinogenic, mutagenic and teratogenic in humans and aquatic organisms.^59^ U causes genetic mutations, lowering of cell reproduction rates and mortality in aquatic animals (*e.g.* fish, crabs); As, Pb, Cd and Cr also bioaccumulate in aquatic organisms, and subsequently in humans *via* consumption, leading to health concerns such as cancer, kidney disfunction and growth impairment in children.^52,59^

With respect to REE recovery, the amounts of the toxic metals (Cr, As, Pb, Cd) recovered in the acid soluble fraction in the fly ash samples studied (ESI Table S2†), were by factors of 10s and 100s, less than the World Health Organisation (WHO) maximum permissible limits in soil.^60^ Also, the amounts of Th and U, which are both radioactive, were by factors of 100s, lower than the natural background level of 1–10 mg kg^−1^. This translates to lower costs of waste handling as the leach solution left behind following selective extraction of the REEs, together with the heap leached fly ash residue, can be recycled into bricks, stabilising/locking the toxic heavy metals in the matrices of the bricks. Recycling the residual fly ash (post heap-leaching) with the waste solution adds extra value, reduces the long-term potential health and environmental hazards posed by the toxic heavy metals, and also reduces the carbon footprint of the process relative to conventional brick and concrete production. However, recycling of heap leaching waste solution and residual fly ash into bricks is dependent on country specific legislation on the permissible concentrations of toxic heavy metals in bricks. Also, the concentration of toxic heavy metals in coal and coal fly ash of different sources vary, and this variation is dependent on the mineralogy and geology of coal and of coal basin.

## 4. Conclusions

This study has determined the distribution and leaching behaviour of REEs and toxic heavy metals in Nigerian coal and simulant coal fly ash, to better understand its resource potential (especially at low temperatures), and to inform management practices to safeguard the environment from potential heavy metal contamination. The following can be concluded:

• EDS maps of REE-bearing monazite microcrystals indicate that the REEs are distributed toward crystal surfaces, while U and Th occur deeper within the grains (*i.e.*, they are depleted within the surface).

• Significant amounts of the REEs (14 wt% to 31 wt%) were recovered from the acid-soluble fraction of the fly ash when leached using ethanoic acid.

• The greatest amounts of REEs were contained in the residual fraction of the fly ash (39 wt% to 68 wt%).

• Very low amounts (∼1 wt%) of REEs were recovered from the acid-soluble fraction of the coal samples.

• In all the coal samples, the toxic heavy metals were most concentrated in the stable oxidisable fraction (53 wt% to 96 wt%) and residual fractions (24 wt% to 54 wt%).

• As (11 wt%), Pb (4 wt%) and Cd (15 wt%) were significantly recovered in the acid-soluble fraction, with very low (∼1 wt%) amounts of Cr, Th and U recovered in the same fraction.

• In the fly ash samples, As (46 wt% to 60 wt%) was the most enriched in the acid-soluble fraction, followed by Cd (15 wt% to 34 wt%).

• While the greatest amounts of U (53 wt% to 62 wt%) and Th (89 wt% to 96 wt%) were only recovered from the intractable residual fraction, small but not insignificant amounts of U were recovered in the acid-soluble fraction from OMA (5 wt%), OKA (3 wt%) and ODA (13 wt%).

The implication of these results for REE recovery from these fly ash samples is that REEs (especially the critical REEs) in the fly ash can be easily and inexpensively recovered using heap-leaching with ethanoic acid, while also reducing the health and environmental hazards of the toxic heavy metals by recycling the residual fly ash (post heap-leaching) into bricks or concrete. This process is potentially suitable for REE recovery from a substantial proportion of the millions of tonnes of fly ash generated by FBC coal-fired power plants globally. However, this process might not be effective for REE recovery from the bottom ash fraction of FBC coal-fired power plants, as bottom ash was not considered in this study.

Further research is recommended to explore cost effective processes for REE separation from leach solutions. This is needed to generate saleable products such as metallic concentrates.

## Funding

The SEM used in this work to conduct imaging and EDS analysis was purchased following funding by the Engineering and Physical Sciences Research Council (EPSRC), (Reference: EP/K040340/1). Also, this research was financially supported by the Research Fund for Coal and Steel (RFCS) from Mining Waste to Valuable Resource: New Concepts for a Circular Economy (MINRESCUE) project (899518).

## Conflicts of interest

There are no conflicts to declare.

## Supplementary Material

RA-012-D2RA02788G-s001
