# Supplementary material for: Characterisation of rare earth elements and toxic heavy metals in coal and coal fly ash
Source: RSC Adv. 2022 Jul 1;12(30):19284–96. doi: 10.1039/d2ra02788g (PMC9249045; doi:10.1039/d2ra02788g)
Supplement: RA-012-D2RA02788G-s001 [file RA-012-D2RA02788G-s001.pdf]

Electronic Supplementary Material (ESI) for RSC Advances.
This journal is © The Royal Society of Chemistry 2022

Characterisation of rare earth elements and toxic heavy metals in coal and coal fly ash

Ilemona C. Okeme [a]*, Richard A. Crane [b], William Mark Nash [b], Theophilus I. Ojonimi [c], Thomas B. Scott [d]

[a,d] School of Physics, University of Bristol, HH Wills Physics Laboratory, Bristol, BS8 1TL, United Kingdom.

[b] Camborne School of Mines, College of Engineering, Mathematics and Physical Sciences, University of Exeter, UK; Environment and Sustainability Institute, University of Exeter, UK.

[c] Department of Mining Engineering, University of Jos, Jos, 930222, Nigeria.

*** Corresponding author**:

Email: ilemona.okeme@gmail.com

Tel: +44 (0)7376625377

Table S1: Absolute values (mean ± standard deviation; in $mg.kg^{-1}$) of REEs in the various sequential extraction fractions for coal samples (OMC, OKC, ODC).

**Sample/ Fractions**	La	Ce	Pr	Nd	Sm	Y	Eu	Gd	Tb	Dy	Ho	Er	Tm	Yb	Lu
**OMC**															
F1	0.01 ± 0.00	0.01 ± 0.00	0.00	0.01 ± 0.00	0.00	0.01 ± 0.01	0.00	0.00	0.00	0.00	0.00	0.00	0.00	0.00	0.00
F2	0.82 ± 0.02	2.37 ± 0.06	0.33 ± 0.01	1.61 ± 0.04	0.33 ± 0.01	2.54 ± 0.07	0.08 ± 0.00	0.43 ± 0.01	0.06 ± 0.00	0.33 ± 0.01	0.06 ± 0.00	0.16 ± 0.01	0.02 ± 0.00	0.10 ± 0.00	0.01 ± 0.00
F3	2.41 ± 0.05	8.37 ± 0.18	1.34 ± 0.03	6.87 ± 0.15	1.77 ± 0.04	10.41 ± 0.26	0.42 ± 0.01	2.02 ± 0.06	0.30 ± 0.01	1.76 ± 0.05	0.35 ± 0.01	0.89 ± 0.03	0.11 ± 0.00	0.65 ± 0.02	0.09 ± 0.00
F4	3.42 ± 0.07	8.00 ± 0.20	0.89 ± 0.02	3.30 ± 0.07	0.61 ± 0.01	1.40 ± 0.03	0.11 ± 0.00	0.49 ± 0.01	0.06 ± 0.00	0.33 ± 0.01	0.06 ± 0.00	0.14 ± 0.00	0.02 ± 0.00	0.09 ± 0.04	0.01 ± 0.00
**Total**	**6.66**	**18.75**	**2.56**	**11.79**	**2.71**	**14.36**	**0.62**	**2.94**	**0.43**	**2.42**	**0.47**	**1.20**	**0.15**	**0.83**	**0.11**
**OKC**															
F1	0.11 ± 0.01	0.17 ± 0.01	0.02 ± 0.00	0.09 ± 0.01	0.01 ± 0.00	0.07 ± 0.01	0.00	0.02 ± 0.00	0.00	0.01 ± 0.00	0.00	0.00	0.00	0.00	0.00
F2	0.71 ± 0.04	1.35 ± 0.10	0.14 ± 0.01	0.56 ± 0.05	0.10 ± 0.01	0.58 ± 0.05	0.03 ± 0.00	0.12 ± 0.01	0.02 ± 0.00	0.01 ± 0.01	0.02 ± 0.00	0.05 ± 0.00	0.01 ± 0.00	0.03 ± 0.00	0.00
F3	4.56 ± 0.10	10.24 ± 0.24	1.23 ± 0.02	5.05 ± 0.13	1.07 ± 0.03	5.45 ± 0.1	0.26 ± 0.01	1.14 ± 0.02	0.17 ± 0.00	0.95 ± 0.02	0.19 ± 0.00	0.49 ± 0.01	0.06 ± 0.00	0.37 ± 0.01	0.05 ± 0.00
F4	2.32 ± 0.08	4.79 ± 0.16	0.52 ± 0.02	1.94 ± 0.08	0.35 ± 0.02	0.94 ± 0.03	0.07 ± 0.00	0.31 ± 0.01	0.04 ± 0.00	0.20 ± 0.00	0.04 ± 0.00	0.09 ± 0.00	0.01 ± 0.00	0.06 ± 0.00	0.01 ± 0.00
**Total**	**7.71**	**16.56**	**1.91**	**7.64**	**1.54**	**7.04**	**0.36**	**1.59**	**0.22**	**1.25**	**0.24**	**0.63**	**0.08**	**0.46**	**0.06**
**ODC**															

F1	0.08 ± 0.01	0.13 ± 0.03	0.01 ± 0.00	0.06 ± 0.01	0.01 ± 0.00	0.09 ± 0.03	0.00	0.01 ± 0.00	0.00	0.01 ± 0.00	0.00	0.00	0.00	0.00	0.00
F2	1.52 ± 0.06	2.93 ± 0.11	0.32 ± 0.01	1.29 ± 0.04	0.23 ± 0.01	1.71 ± 0.05	0.06 ± 0.00	0.29 ± 0.01	0.04 ± 0.00	0.24 ± 0.01	0.05 ± 0.00	0.13 ± 0.00	0.02 ± 0.00	0.08 ± 0.00	0.01 ± 0.00
F3	4.45 ± 0.06	10.43 ± 0.14	1.24 ± 0.01	5.11 ± 0.04	1.07 ± 0.01	6.24 ± 0.10	0.27 ± 0.01	1.16 ± 0.02	0.17 ± 0.00	1.04 ± 0.02	0.21 ± 0.00	0.58 ± 0.01	0.08 ± 0.00	0.45 ± 0.01	0.07 ± 0.00
F4	6.02 ± 0.15	13.81 ± 0.36	1.47 ± 0.04	5.22 ± 0.14	0.91 ± 0.02	1.73 ± 0.04	0.18 ± 0.01	0.70 ± 0.01	0.09 ± 0.00	0.43 ± 0.01	0.07 ± 0.00	0.16 ± 0.01	0.02 ± 0.00	0.10 ± 0.00	0.01 ± 0.00
**Total**	**12.08**	**27.29**	**3.04**	**11.68**	**2.22**	**9.77**	**0.51**	**2.16**	**0.30**	**1.71**	**0.33**	**0.88**	**0.11**	**0.64**	**0.09**

F1: Acid Soluble Fraction; F2: Reducible Fraction; F3: Oxidisable Fraction; F4: Residual Fraction.

OMC: Omelewu Coal

OKC: Okaba Coal

ODC: Odagbo Coal

Table S2: Absolute values (mean ± standard deviation; in mg.kg^{-1}) of toxic heavy metals in the various sequential extraction fractions for both coal and simulant fly ash samples (OMC, OMA, OKC, OKA, ODC and ODA).

**Sample/Fractions**	Cr	As	Pb	Cd	Th	U
**OMC**						
F1	0.15 ± 0.05	0.12 ± 0.04	0.36 ± 0.00	0.01 ± 0.00	0.001 ± 0.00	0.002 ± 0.00
F2	0.19 ± 0.05	0.59 ± 0.03	2.30 ± 0.36	0.02 ± 0.00	0.0003 ± 0.00	0.004 ± 0.00
F3	23.82 ± 0.59	0.42 ± 0.04	4.75 ± 0.17	0.04 ± 0.00	2.69 ± 0.08	1.20 ± 0.04
F4	0.76 ± 0.09	0	1.62 ± 0.09	0.001 ± 0.00	1.85 ± 0.02	0.18 ± 0.00
**Total**	**24.92**	**1.14**	**9.02**	**0.07**	**4.54**	**1.39**
**OMA**						
F1	0.10 ± 0.03	2.53 ± 0.09	0.47 ± 0.22	0.004 ± 0.00	0.007 ± 0.00	0.03 ± 0.01
F2	0.15 ± 0.02	1.03 ± 0.14	0.68 ± 0.15	0.001 ± 0.00	0.001 ± 0.00	0.001± 0.00
F3	0.75 ± 0.27	0.55 ± 0.16	0.68 ± 0.16	0.02.11 ± 0.00	0.12 ± 0.01	0.24 ± 0.02
F4	5.00 ± 0.57	0.27 ± 0.08	0.48 ± 0.13	0.004 ± 0.00	3.09 ± 0.29	0.32 ± 0.03
**Total**	**5.97**	**4.38**	**2.31**	**0.03**	**3.21**	**0.59**
**OKC**						
F1	0.12 ± 0.02	0.39 ± 0.07	0.15 ± 0.07	0.006 ± 0.00	0.002 ± 0.00	0.00
F2	0.15 ± 0.05	0.55 ± 0.02	0.86 ± 0.13	0.007 ± 0.00	0.00	0.00
F3	9.11 ± 0.17	0.49 ± 0.01	3.14 ± 0.12	0.04 ± 0.00	1.25 ± 0.06	0.41 ± 0.01
F4	0.70 ± 0.38	0	0.50 ± 0.03	0.001 ± 0.00	0.70 ± 0.05	0.07 ± 0.00
**Total**	**10.08**	**1.43**	**4.65**	**0.05**	**1.95**	**0.48**
**OKA**						
F1	0.36 ± 0.04	7.46 ± 0.46	0.38 ± 0.09	0.01 ± 0.00	0.01 ± 0.00	0.06 ± 0.01
F2	0.15 ± 0.01	4.79 ± 0.05	0.18 ± 0.07	0.002 ± 0.00	0.0002 ± 0.00	0.004 ± 0.00

F3	0.67 ± 0.08	1.90 ± 0.14	0.60 ± 0.15	0.02 ± 0.00	0.80 ± 0.05	0.67 ± 0.04
F4	11.50 ± 0.90	2.03 ± 0.38	1.84 ± 0.01	0.01 ± 0.00	6.85 ± 0.24	1.23 ± 0.07
**Total**	**12.68**	**16.18**	**2.99**	**0.04**	**7.66**	**1.97**
**ODC**						
F1	0.09 ± 0.00	0.31 ± 0.06	0.33 ± 0.09	0.01 ± 0.7	0.004 ± 0.01	0.003 ± 0.00
F2	0.13 ± 0.02	0.51 ± 0.07	1.53 ± 0.08	0.005 ± 0.00	0.003 ± 0.00	0.003 ± 0.00
F3	12.06 ± 0.16	0.54 ± 0.02	4.76 ± 0.04	0.04 ± 0.00	1.43 ± 0.01	0.47 ± 0.01
F4	0.56 ± 0.01	0.00	1.24 ± 0.02	0.002 ± 0.00	1.61 ± 0.03	0.13 ± 0.004
**Total**	**12.84**	**1.36**	**7.86**	**0.05**	**3.05**	**0.61**
**ODA**						
F1	0.68 ± 0.02	13.21 ± 0.20	0.33 ± 0.15	0.01 ± 0.004	0.012 ± 0.00	0.29 ± 0.00
F2	0.24 ± 0.15	4.65 ± 0.30	0.27 ± 0.05	0.002 ± 0.0.00	0.004 ± 0.00	0.014 ± 0.00
F3	0.74 ± 0.07	2.27 ± 0.19	1.25 ± 0.04	0.02 ± 0.00	0.72 ± 0.03	0.78 ± 0.05
F4	14.90 ± 0.86	2.00 ± 0.10	6.79 ± 0.30	0.01 ± 0.00	6.43 ± 0.41	1.21 ± 0.08
**Total**	**16.56**	**22.14**	**8.64**	**0.04**	**7.16**	**2.29**

OMA: Omelewu simulant fly ash; OMC: Omelewu Coal

OKA: Okaba simulant fly ash; OKC: Okaba Coal

ODA: Odagbo simulant fly ash; ODC: Odagbo Coal
